# DNA Environment of Centromeres and Non-Homologous Chromosomes Interactions in Mouse

**DOI:** 10.3390/cells10123375

**Published:** 2021-12-01

**Authors:** Victor Spangenberg, Mikhail Losev, Ilya Volkhin, Svetlana Smirnova, Pavel Nikitin, Oxana Kolomiets

**Affiliations:** Vavilov Institute of General Genetics, Russian Academy of Sciences, 119991 Moscow, Russia; losev.mi@phystech.edu (M.L.); volkhin2@fbb.msu.ru (I.V.); s.v.smirnova.genet@gmail.com (S.S.); nikitinp@fbb.msu.ru (P.N.); olkolomiets@mail.ru (O.K.)

**Keywords:** prophase I of meiosis, chromatin, chromosome, synaptonemal complex, satellite DNA, chromocenter, nuclear architecture, sex chromosomes, chromatin silencing, MSCI, γH2Ax, interactions of non-homologous chromosomes

## Abstract

Although the pericentromeric regions of chromosomes that are enriched in tandemly repeated satellite DNA represent a significant part of eukaryotic genomes, they remain understudied, which is mainly due to interdisciplinary knowledge gaps. Recent studies suggest their important role in genome regulation, karyotype stability, and evolution. Thus, the idea of satellite DNA as a junk part of the genome has been refuted. The integration of data regarding molecular composition, chromosome behaviour, and the details of the in situ organization of pericentromeric regions is of great interest. The objective of this work was a cytogenetic analysis of the interactions between pericentromeric regions from non-homologous chromosomes in mouse spermatocytes using immuno-FISH. We analysed two events: the associations between centromeric regions of the X chromosome and autosomes and the associations between the centromeric regions of the autosomal bivalents that form chromocenters. We concluded that the X chromosome forms temporary synaptic associations with different autosomes in early meiotic prophase I, which can normally be found until the pachytene–diplotene, without signs of pachytene arrest. These associations are formed between the satellite-DNA-rich centromeric regions of the X chromosome and different autosomes but do not involve the satellite-DNA-poor centromeric region of the Y chromosome. We suggest the hypothetical model of X chromosome competitive replacement from such associations during synaptic correction. We showed that the centromeric region of the X chromosome in association remains free of γH2Ax-dependent chromatin inactivation, while the Y chromosome is completely inactivated. This finding highlights the predominant role of associations between satellite DNA-rich regions of different chromosomes, including the X chromosome. We suppose that X-autosomal transient associations are a manifestation of an additional synaptic disorder checkpoint. These associations are normally corrected before the late diplotene stage. We revealed that the intense spreading conditions that were applied to the spermatocyte I nuclei did not lead to the destruction of stretched chromatin fibers of elongated chromocenters enriched in satellite DNA. The tight associations that we revealed between the pericentromeric regions of different autosomal bivalents and the X chromosome may represent the basis for a mechanism for maintaining the repeats stability in the autosomes and in the X chromosome. The consequences of our findings are discussed.

## 1. Introduction

### 1.1. Pericentromeric Satellite DNA

Pericentromeric satellite DNAs, a distinctive component of eukaryotic genomes, that had been considered “junk”, have evolved over the past few decades to “powerful and active contributors to genomic and chromosomal evolution”, providing genome regulation and stability [1]. Nevertheless, modern genome sequencing methods have a number of limitations due to the difficulties in the assembly and analysis of large blocks of tandemly repeated DNA elements in centromeric regions. Important information about the size, structure, and the role in the interchromosomal interactions of these DNA regions is still understudied.

Murine pericentromeric chromatin that is composed of well-detailed satellite DNA families is of particular interest [2]. In mice pericentromeric, chromatin manifests as large blocks at the proximal ends of the chromosomes [3,4]. Dozens of different DNA satellites have been found in the pericentromeric regions of the *Mus musculus* chromosomes [5]. The two largest satellites, Major satellite (MajSat) and Minor satellite (MiSat), are located in the pericentromeric heterochromatin and represent AT-rich regions [2,6,7]. Fluorescence in situ hybridisation (FISH) probes targeting the monomers of MajSat and MiSat DNA repeats demonstrate strong signals in the centromeric regions of all of the autosomes and the X-chromosome (but not the Y-chromosome) of the *Mus musculus* karyotype [8,9]. According to Guenatri et al. (2004), the MiSat DNA is considered to be associated with centromere functioning, while the MajSat DNA is part of the heterochromatin that is responsible for the aggregation of various cluster-forming chromosomes [10]. In general, the murine telocentric chromosomal domain as a whole exhibits high sequence identity between nonhomologous chromosomes (>98%) [11]. However, the role of MajSat DNA in cell division is still unclear [2].

### 1.2. Chromocenters and Ectopic Contacts of Pericentromeric Satellite DNA in Meiotic Prophase I

The studies that have been performed on meiotic chromosomes have mainly focused on the prophase I nuclei. In particular, researchers have explored the key substages that are associated with homologous chromosome synapsis, double-strand break (DSB) processing, and early and late meiotic recombination events, including crossing over [12,13,14,15,16,17]. In most organisms, centromeric regions in particular and heterochromatic regions in general appear to cluster and decluster dynamically during early meiotic prophase I, suggesting an important—though unclear—role for these processes [12,17,18].

In early meiosis, non-homologous centromere pairing is a usual pattern, and later, it is normally corrected by homologous synapsis and assembly of bivalents [19,20]. This process is connected with the chromosomal “bouquet” structure, where the clustering of the chromosomal telomere ends at a local territory of the inner nuclear envelope and promotes the initiation of synapsis in the eukaryotes [12,17,21,22,23,24,25,26]. As a result, late zygotene and early pachytene nuclei are characterized by the finalization of the homologous synapsis of chromosomes and the complete correction of non-homologous synaptic associations [12].

In pachytene nuclei, due to telomere declustering, the completely assembled bivalents demonstrate arc-shaped “trajectories” through the nuclear space [27]. Numerous cytogenetic studies that have been performed on nuclei passing through the presynaptic [20] and postsynaptic [15,28,29] stages of meiotic prophase I have specific chromatin structures that have been described and that are known as chromocenters, which include pericentromeric satellite DNAs from different chromosomes [15]. Studies of the three-dimensional (3D) reconstruction of murine synaptonemal complex karyotypes (2n = 40) have revealed that these bivalent associations are random [28,29].

### 1.3. Pericentromeric Satellite DNA and Ectopic Recombination

An analysis of animal karyotypes led to the hypothesis that the sequence identity of the pericentromeric regions is connected with the mechanism of frequent recombination exchanges between non-homologous chromosomes [11,30]. The question as to whether such recombination mechanisms have a somatic or meiotic origin of such recombination mechanisms remains unresolved, although data obtained from *Drosophila* point to the predominant role of meiosis [31]. Recombination events between oppositely oriented repeats, including mouse MiSat DNA, are also considered prerequisites for Robertsonian translocations [11]. The authors assumed that a possible recombination mechanism should promote the stable evolutionary maintenance of the telocentric karyotype in mice [11]. A confirmation of the occurrence of DNA exchanges between nonhomologous chromosomes has also been obtained in human and apes for tandemly repeated ribosomal DNA (rDNA) genes (nucleolus organizers, NOR, 28S rDNA) [32]. Subsequent studies have revealed that recombination in the rDNA locus of higher eukaryotes is frequent enough to monitor changes within a few generations [33]. Studies of mouse meiotic nuclei have revealed the association of nucleolus organizer regions (NORs) from different bivalents to form a common nucleolus [27] but could not provide any predictability in other associations of autosomal bivalents.

Possible mechanisms of ectopic recombination are being studied in the context of processing DNA DSB [34,35]. DSB processing (formation and subsequent repair) had been considered impossible in the heterochromatic regions near centromeres, but subsequently, the possibility of the transient relaxation of heterochromatin and DSB processing has been described [36]. DNA DSB repair in the pericentromeric regions of tandemly repeated DNA is particularly challenging because the risk of aberrant recombination is increased in these regions, leading to the loss or duplication of information [30,34,35]. Nevertheless, unequal recombination events have been suggested as a mechanism for the generation and fixation of subtly different higher-order repeats [37]. Later studies have revealed that DSB processing is possible in pericentromeric chromatin, but induced DSBs and γH2AX foci (chromatin inactivation and recruitment of repair factors to damaged DNA) rapidly disappear due to relocation and quick DNA repair [35,38]. Studies considering DSB processing in heterochromatin suggest that single-strand annealing (SSA) as well as non-homologous end joining (NHEJ) mechanisms are more involved in the DNA repair in pericentromeric regions rather than homologous recombination [36,39,40].

The *M. musculus* karyotype, which only consists of telocentric chromosomes, is a remarkable model for studying the dynamics of chromosomal territories and interactions between chromosomes in meiosis [12]. Our work is an integration of the behaviour of chromosomes during meiotic prophase I, an immuno-FISH study of satellite DNA of chromocenters and the inactivation of sex chromosomes in mice. The aims of this study were to investigate the interbivalent chromatin contacts in the mouse meiotic karyotype as well as the associations between the X chromosome and autosomal bivalents, with a special focus on pericentromeric satellite DNA.

## 2. Materials and Methods

### 2.1. Preparation of Synaptonemal Complexes (SCs)

Germ line cells, spermatocytes I, were obtained from two adult male BALB/c and two adult male CBA mice. Seminiferous tubules were isolated and disaggregated in PBS for 5 min. Conventional spreads of primary spermatocytes nuclei were prepared and fixed using the technique developed by Navarro et al. (1981) [41]. Moderate and intense spreading were implemented through the longer cell suspension incubation period (5–10 min) on the surface of 0.2 M sucrose drops and through the addition of 0.05% Triton X-100 in PBS. Poly-L-lysine-coated slides were used for all of the immunofluorescence studies.

### 2.2. Immunostaining Procedure

Slides were washed with phosphate-buffered saline (PBS) and were incubated with primary antibodies that had been diluted in the antibody dilution buffer (ADB: 3% bovine serum albumin (BSA), 0.05% Triton X-100 in PBS) at 4 °C overnight. SCs were detected using rabbit polyclonal antibodies against the SC axial element protein SYCP3 (ab15093, 1:250, Abcam, Cambridge, UK), centromeres were detected using human anti-centromere antibodies ACA (1:500; Antibodies Incorporated 15–234, Davis, CA, USA), and the inactivated chromatin of the sex bivalents was stained with the mouse monoclonal anti-γH2AX antibodies (ab26350, 1:800, Abcam, Cambridge, UK). After washing, the corresponding secondary antibodies that had been diluted in ADB were used: goat anti-rabbit Immunoglobulin (Ig) G, Alexa Fluor 555 (ab150078, 1:500, Abcam, Cambridge, UK), goat anti-rabbit Immunoglobulin (Ig) G, Alexa Fluor 488 (ab150077, 1:500, Abcam, Cambridge, UK), goat anti mouse Immunoglobulin (Ig) G, Alexa Fluor 555 (ab150118, 1:500, Abcam, Cambridge, UK), and goat anti-human Alexa Fluor 488 (A-11013, 1:500, ThermoFischer, Waltham, MA USA). Secondary antibody incubations were performed in a humid chamber at 37 °C for 2 h.

### 2.3. FISH Procedure

In situ hybridization with satellite DNA probes was performed in two rounds after immunostaining using the fluorescently labeled oligonucleotides MajSat-23bp (FAM)—5’ TTCACGTCCTAAAGTGTGTATTT and MiSat-26bp (TAMRA)—5’ AGTTACACTGAAAAACACATTCGTTG (Syntol, Moscow, Russia) following the protocol described by Stepakov et al. (2015) [42]. FISH procedures were performed after the immunostaining with SYCP3 and ACA, as DNA denaturation also destroys protein epitopes and therefore affects antibodyreactivity.

### 2.4. Microscopy

Slides were examined using the Axioimager D1 microscope (Carl Zeiss, Jena, Germany) equipped with the Axiocam HRm CCD camera (Carl Zeiss, Jena, Germany), Carl Zeiss filter sets (FS01, FS38HE, and FS43HE), and the image-processing AxioVision Release 4.6.3. software (Carl Zeiss, Jena, Germany). All preparations were mounted with the Vectashield antifade mounting medium with DAPI (Vector Laboratories, Burlingame, CA, USA).

### 2.5. Analysis of SC Preparation

The determination of meiotic prophase I stages and sex chromosome identification were performed by the analysis of the combination of the basic morphological criteria that were used in the studies of meiotic cells [13,43,44]. Meiotic bivalent lengths were measured using ImageJ software (https://imagej.nih.gov/ij, accessed on 29 November 2021), and the numbers of autosomal bivalents that were associated with the X chromosome were identified. Data on the nuclei along with the chromosomal associations are available in the Supplementary Materials (Figures S1–S4, xls file “SC measurements”). BALB/c mouse spread nuclei preparation were used in the study, and the CBA spread nuclei were used to confirm that X-autosomal associations are normal for both inbred lines (Figures S11 and S12).

## 3. Results

### 3.1. Associations of X Chromosome with Different Autosomal Bivalents

**Figure 1:**
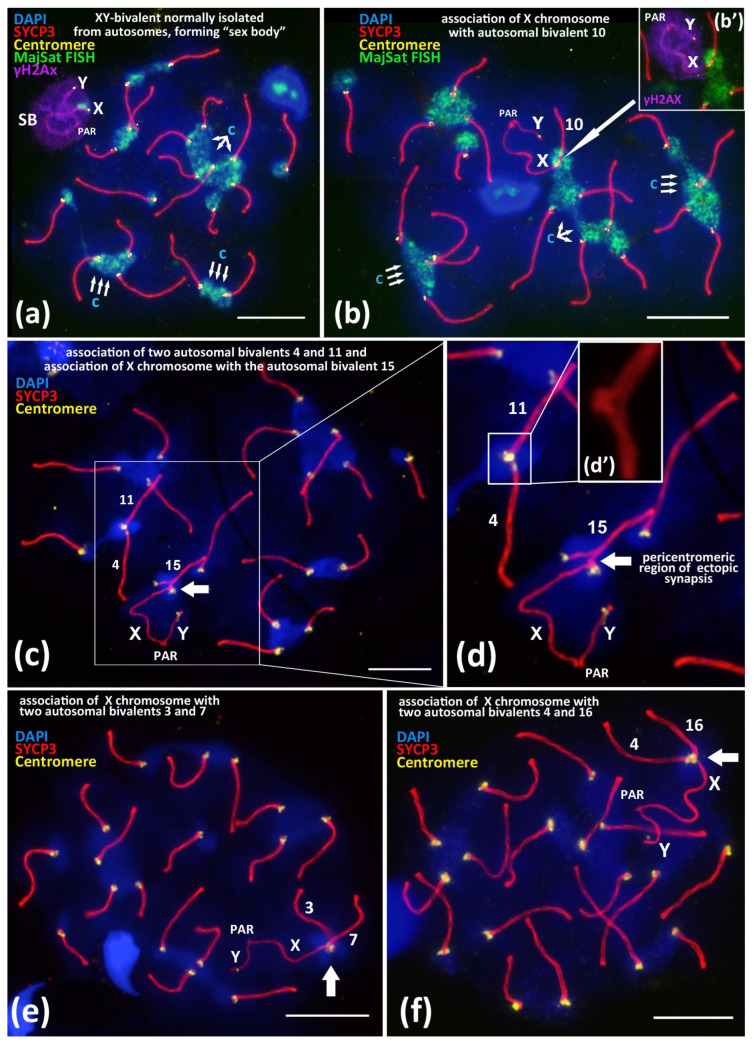
Associations between centromeric regions of autosomal chromosomes and the X chromosome in BALB/c mouse meiotic nuclei. Chromatin was stained with DAPI (blue), axial elements of meiotic chromosomes were immunostained with the antibodies against the SYCP3 protein (red), centromeres were stained with the ACA antibodies (yellow), and inactivated chromatin of asynaptic sex bivalent was immunostained with the antibodies against γH2AX (violet). FISH with oligo-DNA probes to MajSat DNA (green). Pseudoautosomal region of the sex bivalent is indicated as “PAR”; chromocenters are indicated “c”. Scale bar—10 μm. (**a**)—Normal meiotic behavior of XY bivalent forming dense DNA-protein structure “sex body” (SB) that is spatially isolated from autosomes in pachytene. Sex bivalent is “covered” by γH2AX immunostaining (violet), indicating chromatin inactivation. (**b**)—Association between the X chromosome and autosomal bivalent 10 (white arrow). MajSat DNA of X chromosome is included in the chromocenter (green) formed by several autosomal bivalents. (**b’**)—Immunostaining of sex bivalent with anti-γH2AX antibodies (violet) is located on the XY bivalent only but not on the assembled autosomal bivalent 10. (**c**,**d**)—Association of the X chromosome with autosomal bivalent 15 (arrow) and (**d’**)—details of association between two autosomal bivalents 4 and 11. (**e**)—Association of the X chromosome with two autosomal bivalents. 3 and 7. (**f**)—Association of the X chromosome with two autosomal bivalents 4 and 16.

We studied spread preparations of mouse meiotic prophase I nuclei, that is, the total preparations of the synaptonemal complexes. In 95–96% of the pachytene–early diplotene nuclei, the sex bivalent, XY, which normally forms a specific structure, namely the “sex body”, which is spatially isolated from all of the autosomal bivalents. We have confirmed the “sex body” formation by using a well–known marker, the phosphorylated form of histone H2AX (γH2AX) (Figure 1a) [45,46].

**Figure 2:**
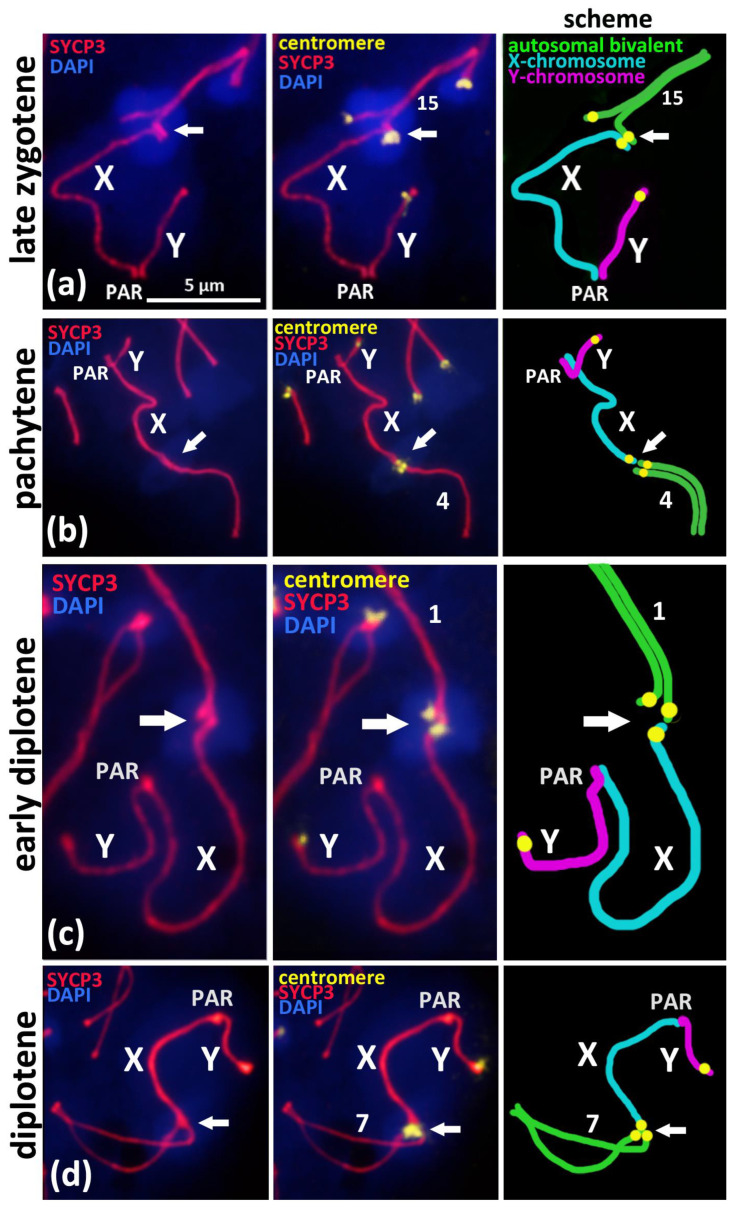
Associations of X chromosome with different autosomal bivalents on the successive stages of meiotic prophase I in BALB/c mouse. Chromatin was stained with DAPI (blue), axial elements of meiotic chromosomes were immunostained with the antibodies against the SYCP3 protein (red), and centromeres were stained with the ACA antibodies (yellow). Pseudoautosomal region of sex bivalent is indicated “PAR”. (**a**)—Late zygotene, short synaptonemal complex (white arrow) assembled “side-by-side” between the pericentromeric regions of the autosomal univalent 15 and the X chromosome. (**b**)—Pachytene, “end-to-end” association of autosomal bivalent 4 and the X chromosome after its competitive replacement from the non-homologous SC-assembly. (**c**)—Early diplotene, “end-to-end” association of the X chromosome and autosomal bivalent 1. (**d**)—Mid-diplotene, “end-to-end” association of the X chromosome and autosomal bivalent 7.

Nevertheless, besides the normal “isolated” location of the sex bivalent from the autosomal bivalents (Figure 1a), we revealed rare (5.6–6.1%) nuclei with non-homologous end-to-end associations between the X chromosome and autosomal bivalents (Figure 1b). Such associations mostly were detected in the pachytene nuclei and less often in the diplotene nuclei (Supplementary Materials, Figures S1–S4), which involve centromeric regions of the X chromosome and the centromeric region of one lateral element of an autosomal bivalent (Figure 1b–d and Figure 2). We revealed that these associations are not bivalent-specific but instead could occur between the X chromosome and different autosomal bivalents (Figure 2). In the Supplementary Materials, we present detailed data of the measured synaptonemal complex (SC) karyotypes and the indicated bivalent numbers (Figures S1–S4, xls-file ‘SC measurements’). Thus, after measurements of the SC-karyotypes without bivalent overlap, we found associations between the X chromosome and almost all of the bivalents of the mouse SC-karyotype: 1, 2, 3, 4, 5, 6, 7, 8, 9, 10, 11, 12, 13, 14, 15, 16, 18, and 19. Important additional findings are the sporadic end-to-end associations of the autosomal bivalents (Figure 1c–d’; Figure S1d) and the extremely rare end-to-end associations between the X chromosome together with two autosomal bivalents. We found two nuclei with associations: X + 3 + 7 (Figure 1e) and X + 4 + 16 (Figure 1f) (Table 1).

Of note, that pericentromeric satellite DNA of the X chromosome and satellite DNA of the autosomal bivalents formed a joint chromocenter, which was visualized by FISH with a MajSat DNA probe (Figure 1b). After a detailed analysis of the preparations from two inbred mouse lines, we found that 6.1% (BALB/c) and 5.6% (CBA) of nuclei, respectively, have such chromosomal associations (Table 1).

To study the mechanism of the associations between the X chromosome and different autosomal bivalents, we traced such cases during the successive stages of meiotic prophase I, which reflect the progression of chromosomal synapsis and desynapsis: late zygotene, pachytene, early diplotene, and mid-diplotene (Figure 2). Thus, in the late zygotene–early pachytene nuclei, we detected the formation of a short synaptonemal complex (white arrow) that was assembled side-by-side between the centromeric regions of an autosomal univalent and the X chromosome in the seven nuclei (Figure 1d or Figure 2a). In pachytene nuclei, we detected the complete synapsis of all of the autosomal bivalents and the end-to-end associations of one of these bivalents with the X chromosome in 32 nuclei (Figure 2b). In the early diplotene and mid-diplotene nuclei, we detected the partial desynapsis of the autosomes (desynaptic forks), the chiasmata and end-to-end associations of the X chromosome, and the partially desynapsed autosomal bivalent in 18 nuclei (Figure 2c,d). We also detected six nuclei with disrupted associations between the X chromosome and the autosomal bivalents in pachytene–diplotene (Supplementary Materials, Figure S5a,b).

### 3.2. Meiotic Silencing of Sex Chromosomes and X-Autosomal Associations

**Figure 3:**
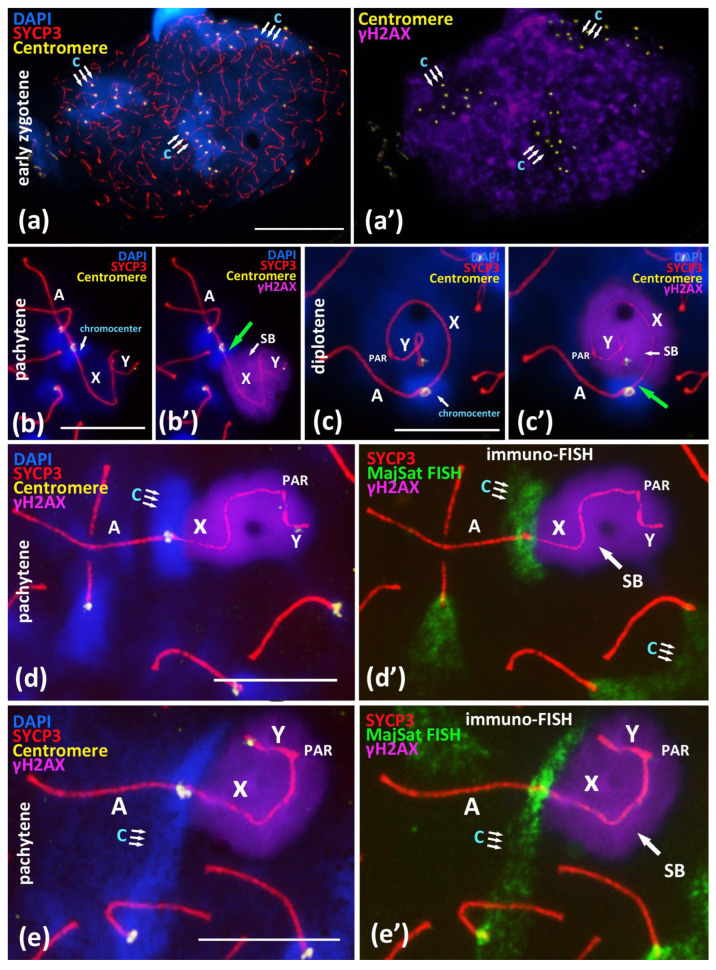
Immuno-FISH study of chromocenters and γH2AX-dependent chromatin inactivation in early zygotene and in the associations between X chromosome and autosomal bivalents in pachytene, BALB/c mouse. Chromatin was stained with DAPI (blue), axial elements of meiotic chromosomes were immunostained with the antibodies against the SYCP3 protein (red), centromeres were stained with the ACA antibodies (yellow), and inactivated chromatin was immunostained with the antibodies against γH2AX. FISH with oligo-DNA probes to MajSat DNA (green). Scale bar—10 μm. (**a**)—Early zygotene spread nucleus with 3 DAPI-rich chromocenters (‘c’, triple arrows) composed of clustered pericentromeric regions of chromosomes; immunostained centromeres (40 yellow foci). Axial elements of asynapsed chromosomes (red). (**a’**)—Expansive γH2AX immunostaining on the same nucleus do not cover three DAPI-rich regions of chromocenters. (**b**,**c**)—Association of X chromosome and autosomal bivalent resulted in the formation of joint chromocenter. (**b’**,**c’**)—Immunostaining revealed that the DAPI-rich pericentromeric region of X chromosome is free of γH2AX (green arrows) in the X-autosomal association. (**d**)—Immuno-FISH study of association of the X chromosome and the autosomal bivalent resulted in the formation of the joint chromocenter (‘c’, triple arrows). (**d’**)—Chromatin region of MajSat (green) and the inactivated chromatin of the “sex body” (violet, γH2AX) are spatially separated. (**e**)—Immuno-FISH study of association of the X chromosome and the autosomal bivalent resulted in the formation of joint chromocenter (‘c’, triple arrows) between two autosomal bivalents and X chromosome. (**e’**)—Chromatin region of MajSat (green) and the inactivated chromatin of the “sex body” (violet, γH2AX) are spatially separated.

We performed a detailed immuno-FISH study of the relationships between chromocenters and γH2AX-dependent chromatin inactivation in early zygotene nuclei and in the chromosomal associations in pachytene and diplotene nuclei (Figure 3). The study of the pericentromeric regions of the chromosomes at the early zygotene stage showed that all of the large chromocenters (Figure 3a,a’), which are the regions where centromere clustering take place, do not undergo explicit γH2AX-dependent chromatin inactivation. γH2AX immunostaining is distributed over the chromatin of yet asynapsed chromosomes and is absent or very weak in DAPI-rich pericentromeric chromatin (Figure 3a,a’).

During the later stages of meiotic prophase I in the pachytene and diplotene nuclei, we studied the γH2AX-inactivated chromatin areas of the sex bivalent and the MajSat DNA of the proximal chromocenter, including the pericentromeric chromatin of both the X chromosome and the autosomal bivalent in association. We revealed that the X-autosomal association, the pericentromeric region of the X chromosome enriched with MajSat DNA, was not immunostained with γH2AX. This region stands out from the “sex body” (Figure 3b–c’).

We detected this pattern in all 26 meiotic nuclei with the combined SYCP3, ACA and γH2AX immunostaining (Figure 3b’,c’; Supplementary Materials Figure S8).

Additional immuno-FISH study revealed that the inactivated chromatin of the “sex body”, which had been immunostained with the anti-γH2AX antibodies, and the chromocenter, which combines the pericentromeric chromatin of the X chromosome and the associated autosomal bivalent, are clearly separated (Figure 3d–e’). That is, they represent spatially separated chromatin domains. We showed that the meiotic sex chromosome inactivation (MSCI) mechanism is not introduced in the pericentromeric satellite DNA-rich region of the X chromosome in the X-autosomal chromocenters.

### 3.3. Peculiarities of Morphology of Chromocenters in Meiosis

**Figure 4:**
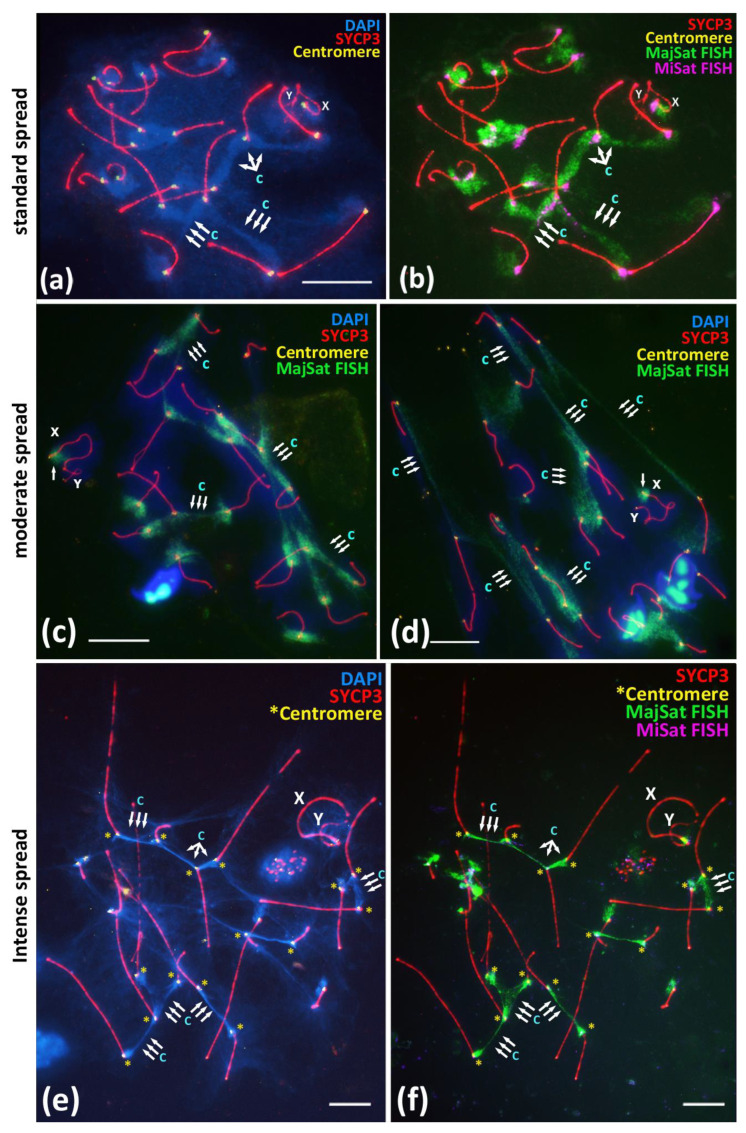
Immuno-FISH study of the Major and Minor satellite DNA localization in the pachytene nuclei spread preparations under different spreading conditions, BALB/c mouse. Chromatin was stained with DAPI (blue), FISH with oligo-DNA probes to MajSat DNA (green), and MiSat DNA (violet). Axial elements of meiotic chromosomes were immunostained with the antibodies against the SYCP3 protein (red), and centromeres were stained with the ACA antibodies (yellow). DAPI-enriched chromatin of chromocenters are indicated “c” and triple arrows. Sex chromosomes are indicated as “X” and “Y”. Scale bar—10 μm. (**a**,**b**)—Immuno-FISH on meiotic prophase I nuclei, pachytene, normal spread. Interbivalent chromocenters demonstrate predominant coverage by MajSat DNA (green) and smaller elongated signals of MiSat DNA (violet). (**c**,**d**)—Immuno-FISH on meiotic prophase I nuclei, pachytene, moderate spread. Chromocenters manifest as stretched chromatin fibers enriched in MajSat DNA. Sex bivalent form spatially separated structure and not connected with autosomal bivalents, the MajSat region of X chromosome is not stretched. (**e,f**)—Immuno-FISH, pachytene, intense spread. Tight linkage of centromeres by interbivalent stretched chromatin fibers. **(e)**—Chromatin of chromocenters is DAPI-enriched and indicated as “c”. (**f**)—The same cell after FISH. Chromocenter chromatin fibers (green) binding the centromeric ends (yellow asterisks) of different autosomal bivalents are enriched in MajSat DNA.

Both the residual associations between the centromeric regions of the non-homologous chromosomes, including the X chromosome (Figure 1b’,d, Figure 2 and Figure 3b–e), as well as the interbivalent connections by the chromocenters (Figure 1a,b or Figure 3a,a’) in prophase I are initiated in early meiosis by centromere clustering [20]. Thus, after detailing the X-autosomal associations, we analyzed more general associations, namely the interbivalent chromatin structures—chromocenters. To analyze the morphology of satellite DNA-rich chromocenters in meiotic prophase I, we performed an immuno-FISH study of the MajSat and MiSat DNA localization in meiotic prophase I nuclei under different spreading conditions in 114 pachytene nuclei. We obtained normal, moderate, and intensive chromatin spreading, resulting in the two-fold spreading of pachytene nuclei (Figure 4).

The normally spread nuclei demonstrated the expected smooth distribution of the pachytene nuclei chromatin similarly to that described in other studies [15,27,28]. All of the assembled synaptonemal complexes could be identified, and the chromocenters were distinguishable as DAPI-enriched regions between the centromeric regions of different bivalents (Figure 4a). The immuno-FISH approach confirmed that the MajSat DNA demonstrated the predominant coverage of the chromocenters regions of the autosomal bivalents and the pericentromeric region of X-univalent in all of the 71 nuclei that were studied (Figure 4b). MajSat DNA signals on the autosomal bivalents overlap the DAPI-enriched chromocenters (Figure 4a,b; Supplementary Materials, Figure S8a–b’). MiSat DNA signals were located near the immunostained centromeres on all bivalents but not on the Y chromosome, a finding similar to the results of other studies [6,7,8] (Figure 4b; Supplementary Materials Figure S7).

Moderate spreading allowed us to examine the wider distribution of the chromatin of the pachytene and diplotene nuclei over the glass surface (Figure 4c,d; Supplementary Materials, Figure S9a–d). Immuno-FISH with oligonucleotide probes targeted to MajSat DNA revealed the elongation of the chromocenters and the formation of stretched chromatin fibers enriched in MajSat DNA. Notably, in all 23 of the nuclei that were studied, no gaps of such interbivalent chromatin fibers mediated by satellite-enriched DNA were detected.

Intensely spread nuclei (20 nuclei studied) demonstrated not a smooth but instead an irregular and stretched chromatin structure, most of which comprised linear axial elements of SCs (bivalents) that radially diverged from the center of the preparation or that stretched in one direction (Supplementary Materials, Figures S9e,f and S10). The DAPI-enriched chromatin territories and the chromocenters demonstrated “cord-like” structures due to the intense spreading (Figure 4e). Immuno-FISH showed that both MiSat and MajSat DNA were detected as the stretched linear interbivalent signals (Figure 4f; Figures S7, S9e–f and S10).

Overall, we showed that both MajSat and MiSat DNAs are involved in the formation of interbivalent chromatin structures, which are surprisingly stable at the pachytene and diplotene stages of meiotic prophase I.

## 4. Discussion

### 4.1. Chromocenters and Associations between Non-Homologous Chromosomes in Meiosis

It is known that in the early stages of meiotic prophase I (leptotene and zygotene), the formation of the specific chromosomal “bouquet” occurs, i.e., clustering of telomere ends of meiotic chromosomes [23,26]. Scherthan (2001) wrote that during “bouquet” formation, “most terminal chromosome fractions are aligned, allowing intensive chromatin interactions” [23]. Later, in the late zygotene and in the early pachytene nuclei, the telomere ends of the assembling bivalents begin to decluster and become evenly distributed over the inner nuclear envelope of the mid-pachytene nuclei [23]. Thus, the chromosomal “bouquet” provides the initiation of the contacts of telomeric ends in leptotene nuclei, promotes the correction of synaptic errors (non-homologous synapsis) during the zygotene stage, and results in residual associations between the bivalents in the postsynaptic stages of meiotic prophase I (Supplementary Materials Figure S6) [12,19,20]. Previous studies of meiotic nuclear architecture in mouse models have revealed that after the chromosomal “bouquet” stage, the declustering of bivalent ends leads to the formation of smaller “aggregates”, chromocenters between the bivalents at the late zygotene and pachytene stages; these structures remain stable until the later stages of meiotic prophase I [20,29]. Studies using mathematical models suggest random, non-specific associations between bivalents in murine meiotic nuclei [27,28]. This view is consistent with our immuno-FISH data (Figure 1a–f; Figures S1–S4).

### 4.2. Sex Chromosomes Behaviour and Chromatin Inactivation during Meiotic Prophase I

The analysis of the sex bivalent behaviour during meiosis is of particular importance. In mice, the centromeric regions of the X and Y chromosomes do not synapse during meiotic prophase I (Figure 1a) [45,46,47]. The only region of partial synapsis (pseudoautosomal region, PAR) on the sex bivalent is located at the distal regions of the X and Y [48]. It is also known that the XY bivalent demonstrates distinct behaviour compared to the autosomal bivalents in meiotic prophase I in mice [45,49]. This manifests in the delay of key events such as synapsis and DSB repair [50,51]. In addition, sex chromosomes that have been subjected to silencing during meiotic prophase I by a mechanism called meiotic sex chromosome inactivation (MSCI) leads to the formation of the “sex body”, a dense DNA–protein structure that covers the sex bivalent [45,46,49,52]. Thus, during the pachytene and diplotene stages, the sex bivalent demonstrates the morphological features of the processes that were initiated earlier in the leptotene–zygotene but that were not completed due to the limited region of homology and meiotic sex chromosome inactivation MSCI [45,51].

### 4.3. Detailing the Mechanism of Associations between the X Chromosome and Different Autosomal Bivalents in Meiotic Prophase I

We analyzed the X-autosomal associations on the successive stages of meiotic prophase I (Figure 1b–f, Figure 2 and Figure 3). Late zygotene nuclei with X-autosomal associations demonstrated the side-by-side assembly of short synaptonemal complexes between the pericentromeric region of the X chromosome and the asynaptic region of autosomal bivalent (Figure 2a). This important finding highlights that the initial mechanism of such associations is the meiotic synapsis—pericentromeric assembly of short synaptonemal complexes. Normally, local non-homologous synaptic regions should be corrected during the zygotene stage and should be completely corrected before the pachytene stage starts [20]. Here, we detected a delay in the correction of such ectopic synaptic regions.

In the pachytene and diplotene stages, the end-to-end associations between the X chromosome and one of the autosomal chromosomes (Figure 2b–d) are most likely the result of the competitive replacement of the X chromosome by the second homologous autosome to form the completely assembled synaptonemal complex (Figure 2b–d). The fact that the X chromosome is attached to only one chromosome from the ectopically associated autosomal bivalent also supports our view (Figure 2a–c; Figure S4f). Thus, we suppose that such associations between the X chromosome and autosomal bivalents that we often detected in pachytene and diplotene nuclei are the result of “unfinished synaptic corrections” of random associations between centromeric regions of the X chromosome and the autosomes that are initiated in early meiosis.

It is known that non-homologous chromosomal associations normally occur in early meiosis and have been well detailed in animals [53,54] and plants [55]. Recently, Kasemi and Taketo (2021) [17] revealed that in the SC-karyotype of the BALB/c mice, the centromeric regions remain in an asynaptic state longer than the distal chromosome ends in late zygotene nuclei do [17]. This phenomenon is consistent with our results (Figure 1c–d) and could explain the preservation or formation of non-homologous synaptic associations, namely those between the centromeric regions of autosomes and the X chromosomes.

According to our results, the associations between the X chromosome and an autosomal bivalent is retained until the pachytene–diplotene stages in 5.6–6.1% of cells (Table 1). It cannot be ruled out that such associations of the X chromosome with the autosomes are a manifestation of the “controlling” role of the X chromosome. It is known that in spermatocytes of sterile males that are heterozygous for chromosomal translocations, the disruption of chromosome synapsis and incomplete DSBs repair in meiotic prophase I leads to the so-called pachytene arrest of meiosis [56]. This phenomenon is expressed morphologically in the association of the XY bivalent with heteromorphic SC-bivalents or SC-multivalents, in which synapsis and DSBs DNA repair are impaired [52,56,57]. In this case, the sex bivalent does not migrate to the periphery of the nucleus (i.e., does not form the ‘sex body’) but instead “anchors” among the autosomal SCs [57]. In mice, pachytene arrest is normally observed in 3–5% of pachytene nuclei [58]. The detailed analysis of such associations has resulted in the idea that non-homologous synapsis between asynaptic autosomes and the X-chromosome is a possible way to escape the pachytene checkpoint [56,59]. Our findings of the nuclei with resolved ectopic associations between the X chromosome and autosomes in the pachytene–diplotene indicate the possibility of correcting the X-autosomal associations under study before metaphase I, thus avoiding the arrest and subsequent apoptosis of such cells (Supplementary Materials, Figure S5a,b).

Based on our findings, we can designate the successive stages of correction of ectopic pericentromeric synapsis between the X chromosome and the autosomes (Figure 2a–d) during meiotic prophase I as follows:

1. Ectopic SC assembly. Non-homologous local pericentromeric side-by-side synapsis of the X chromosome and an autosomal univalent. This stage occurs during early meiosis and is characterized by the incomplete assembly of the autosomal bivalent, i.e., asynaptic fork at the pericentromeric region (Figure 2a);
2. Correction of non-homologous synaptic X-autosomal association. This stage includes the complete assembly of the autosomal bivalent and the competitive replacement of the X chromosome from the short non-homologous SC-region (Figure 3b,c). Consequently, the X chromosome remains associated but end-to-end with only one autosomal chromosome (Figure 3b,c);
3. Residual end-to-end association of the X chromosome with the centromeric region of the assembled autosomal bivalent. Such associations can persist until the diplotene stage (Figure 3d) and could also be disrupted before the end of prophase I (Supplementary Materials Figure S5a,b).

### 4.4. Formation of X-Autosomal Chromocenters and MSCI

The most interesting question is how meiotic sex chromosome inactivation (MSCI) acts in the case of ectopic associations with autosomes. As we highlighted above, sex chromosomes in mammals normally form the “sex body”, the dense DNA–protein structure that should protect the sex bivalent from any type of ectopic associations [45,49]. We demonstrated the deviations from this rule, normally occurring in 6.1% of meiotic prophase I nuclei on the postsynaptic stages in BALB/c males (Table 1).

An important aspect of the non-homologous chromosomal associations we described are the contacts between the pericentromeric satellite DNA of the X chromosome and the chromocenters that include the pericentromeric satellite DNA of one or several autosomal bivalents (Figure 1b or Figure 3d’,e’; Figure S7). We detailed the involvement of pericentromeric DNA of the X chromosome in different autosomal chromocenters (Figure 1b or Figure 3d’,e’) and localized the inactivated chromatin (γH2AX) of the “sex body”. Surprisingly, our results showed that the MajSat DNA-rich pericentromeric region of the X chromosome is located outside of the γH2AX immunostaining of the “sex body” (Figure 3d’,e’; Figure S7). Thus, the chromocenter, which includes pericentromeric satellite DNA of the autosomes and X chromosome, is spatially separated from the inactivated chromatin of the “sex body” despite the obligatoriness of the MSCI mechanism during the pachytene stage. This result indicates the great importance of interbivalent contacts of pericentromeric DNA in meiosis. Thus, in the hierarchy of the two processes, the non-homologous contacts of pericentromeric chromatin (chromocenters) and MSCI, the chromocenters but not MSCI occupy the primary role. In the X-autosomal associations under study, we assume the primary role of interbivalent chromocenter formation, but not γH2AX-dependent chromatin inactivation. In addition, MSCI is considered part of a more general mechanism called the meiotic silencing of unsynapsed chromatin (MSUC) [45]. In pachytene nuclei, we observed the complete synapsis of all autosomal bivalents and end-to-end X-autosomal associations, the MajSat DNA of the X chromosome, as part of the X-autosomal temporary chromocenter, which may be under the control of the autosomal MSUC mechanism but not MSCI.

Previous MajSat and MiSat DNA studies in the mouse karyotype [11] as well as our data confirm their localization in the pericentromeric regions of all autosomes and in the X chromosome, but not in the Y chromosome (Figure 1a,b or Figure 4b; Supplementary Materials, Figures S7 and S8). Thus, the Y chromosome is the only chromosome in the *M. musculus* karyotype that cannot undergo homologous meiotic synapsis in its pericentromeric region throughout generations. In addition, our results demonstrated that the centromeric region of the Y chromosome without both satellite DNAs is not involved in chromocenter formation with autosomes in zygotene–diplotene (Figure 1, Figure 2 and Figure 3; Supplementary Materials Figures S1–S4). This fact may be connected with the preservation of autosome-like satellite DNA on the centromeric region of the X chromosome but not on the Y chromosome.

### 4.5. Peculiarities of Chromocenters in Meiosis

The results that we obtained by using three types of spreading of meiotic nuclei, normal, moderate, and intense, demonstrated that the significant part of the “stretched chromatin fibers” connecting the centromeric regions of autosomal bivalents is occupied by the MajSat DNA (Figure 4). Even the intense stretching of chromatin did not lead to the destruction of the chromocenters (Figure 4f; Supplementary Materials Figures S9e,f and S10). It is logical to assume not only colocalization but also the tight contacts of satellite DNA from non-homologous chromosomes in the chromocenters. Taken together, our findings of the X-autosomal interactions, the absence of inactivation of the MajSat DNA of the X chromosome, and the associations between the pericentromeric regions of non-homologous chromosomes (i.e., formation of chromocenters) previously characterized as random [27,28] and characterized by us as very tight, represent new data in favour of the hypothesis of ectopic recombination during meiotic prophase I, a phenomenon that had been proposed previously [11,30,31]. It was shown that suppression of the DSB processing complex could promote alternative SSA, NHEJ, and alt-EJ pathways [11,36,40]. In addition, recent studies suggest that DSB processing is possible in pericentromeric chromatin, but the induced DSBs and γH2AX foci rapidly disappear due to relocation and prompt repair [35,38,40]. This view is consistent with the absence of or very weak γH2AX immunostaining in the chromocenters in early zygotene nuclei (Figure 3a,a’). Below, we list our results in the context of the findings reported by other authors in favour of this hypothesis [11,30,31].

Random interbivalent contacts that have been reported previously [27,28] and ectopic recombination mechanisms [11,30] could allow the uniformity of satellite DNA composition to be maintained between autosomes [11] and X chromosome (Figure 1b, Figure 2 and Figure 3b–e’; Supplementary Materials, Figures S7 and S8b,c).

- We have revealed that the pericentromeric chromatin of the X chromosome may be involved in ectopic interactions with the chromocenters of different autosomes (we detected 18 of 19 bivalents in such associations in our study) (Figure 1b–f and Figure 2; Figures S1–S5). This could be the basis for a mechanism for maintaining autosome-like DNA-repeat composition near the centromere of the X chromosome.
- We have shown that the pericentromeric chromatin of the X chromosome is spatially separated from the inactivated chromatin of the “sex body” (Figure 1b or Figure 3d’,e’; Figure S8). Thus, XY-chromatin inactivation (MSCI) does not interfere with the ectopic X-autosomal interactions of pericentromeric DNAs. This result indicates the high importance of chromocenters formation and functioning during meiosis.
- We have shown that the two-fold stretching of the meiotic nuclei chromatin on the glass surface does not destroy elongated chromocenters (Figure 4a–f; Figures S9 and S10). These “interbivalent chromatin fibers” contain highly repeated MajSat and MiSat DNA of pericentromeric regions of non-homologous chromosomes (Figure 4; Figures S7, S9 and S10). This finding is consistent with the data that centromere but not telomere clustering is the general and primordial meiotic mechanism in eukaryotes [26].

Overall, the interbivalent associations that are mediated by chromocenters are apparently an important mechanism for maintaining the stability of the pericenromeric satellite DNA composition. In our opinion, MajSat DNA plays an important role in this process in mice that provides dynamic stability for the chromosomal territories in meiosis. The important and still unresolved question is whether the interchromosomal connections of satellite DNA are temporary, i.e., stage-specific and could be formed several times during cell cycle or they are constitutive, but not easily detected at certain stages. Indeed, chromatin “bridges” between chromosomes were observed in the interphase and even in the mitotic metaphase plate preparations, but these have still not been generalized for the entire cell cycle [60,61,62].

## 5. Conclusions

1. We suggest the hypothetical model of associations between satellite DNA-enriched regions of autosomes and the centromeric region of the X chromosome. We have detailed the successive stages of correction of these non-homologous X-autosomal associations: early meiotic ectopic synaptonemal complex assembly followed by the competitive replacement of the X chromosome by autosome in later stages (Figure 2a–d).
2. In the case of the X-autosomal association, the pericentromeric region of the X chromosome is integrated in the autosomal chromocenters enriched in MajSat DNA. The centromeric region of the X chromosome is free of γH2Ax-dependent chromatin inactivation (MSCI). Thus, the “sex body” and the proximal X-autosomal MajSat-rich chromocenter are spatially and functionally separated.
3. Our results demonstrate the stability and remarkable sturdiness of the interbivalent chromatin fibers connecting the centromeric regions and that are enriched in satellite DNA in meiotic prophase I nuclei.

The stable interbivalent associations we have detailed, the absence of chromatin inactivation in the X-autosomal chromocenters, and the known data on the DSB processing pathways in heterochromatin [35,38] represent new data that are in favor of a mechanism that is able to maintain pericentromeric repeat stability in autosomes and in the X chromosome [30] as well as insights into the independent evolution of the centromeric DNA content in autosomes and the X chromosome on one hand and in the Y chromosome on the other.

## Figures and Tables

**Table 1:** Associations between X chromosome and autosomal bivalents in two inbred mouse strains.

Strain	X-Autosomal Associations *N* (%)	X-Two Autosomes Associations * *N*	Number of Nuclei Studied
BALB/c	57 (6.1%)	2	935
CBA	48 (5.6%)	0	855

*N*—is the number of nuclei with association cases detected. *—rare associations of X with two autosomal bivalents presented on Figure 1e,f.

## Data Availability

The following information was supplied regarding data availability: Victor Spangenberg. (2021). SC measurements [Data set]. Zenodo. https://doi.org/10.5281/zenodo.5729450 (accessed on 31 October 2021).

## Supplementary Materials

The following are available online at https://www.mdpi.com/article/10.3390/cells10123375/s1, Figures S1–S4: Associations between centromeric regions of autosomal chromosomes and the X chromosome in BALB/c mouse meiotic nuclei, Figure S5: Disrupted associations between centromeric regions of autosomal chromosomes and the X chromosome in BALB/c mouse meiotic nuclei in pachytene and diplotene, Figure S6: Chromocenters in early zygotene and pachytene in BALB/c mouse, Figure S7: Immuno-FISH with oligo-DNA probes to Minor Satellite DNA in the spread preparation of meiotic nucleus BALB/c mouse, Figure S8: Immuno-FISH identification of MSCI in the associations between Major satellite DNA of the X chromosome and autosomal chromocenters, Figures S9 and S10: “Immuno-FISH study of the Major satellite DNA localization in the pachytene nuclei spread preparations under different spreading conditions, BALB/c mouse”, Figures S11 and S12: Associations between centromeric regions of autosomal chromosomes and the X chromosome in CBA mouse meiotic nuclei.
