# Supplementary material for: DNA Environment of Centromeres and Non-Homologous Chromosomes Interactions in Mouse
Source: Cells. 2021 Dec 1;10(12):3375. doi: 10.3390/cells10123375 (PMC8699862; doi:10.3390/cells10123375)
Supplement: Supplementary file 1 [file cells-10-03375-s001.zip › cells-1466122 Supplementary Materials Spangenberg et al 2021.pdf]

DNA environment of centromeres and non-homologous chromosomes interactions in mouse

Victor Spangenberg, Mikhail Losev, Ilya Volkhin, Svetlana Smirnova, Pavel Nikitin, Oxana Kolomiets

SUPPLEMENTARY MATERIALS

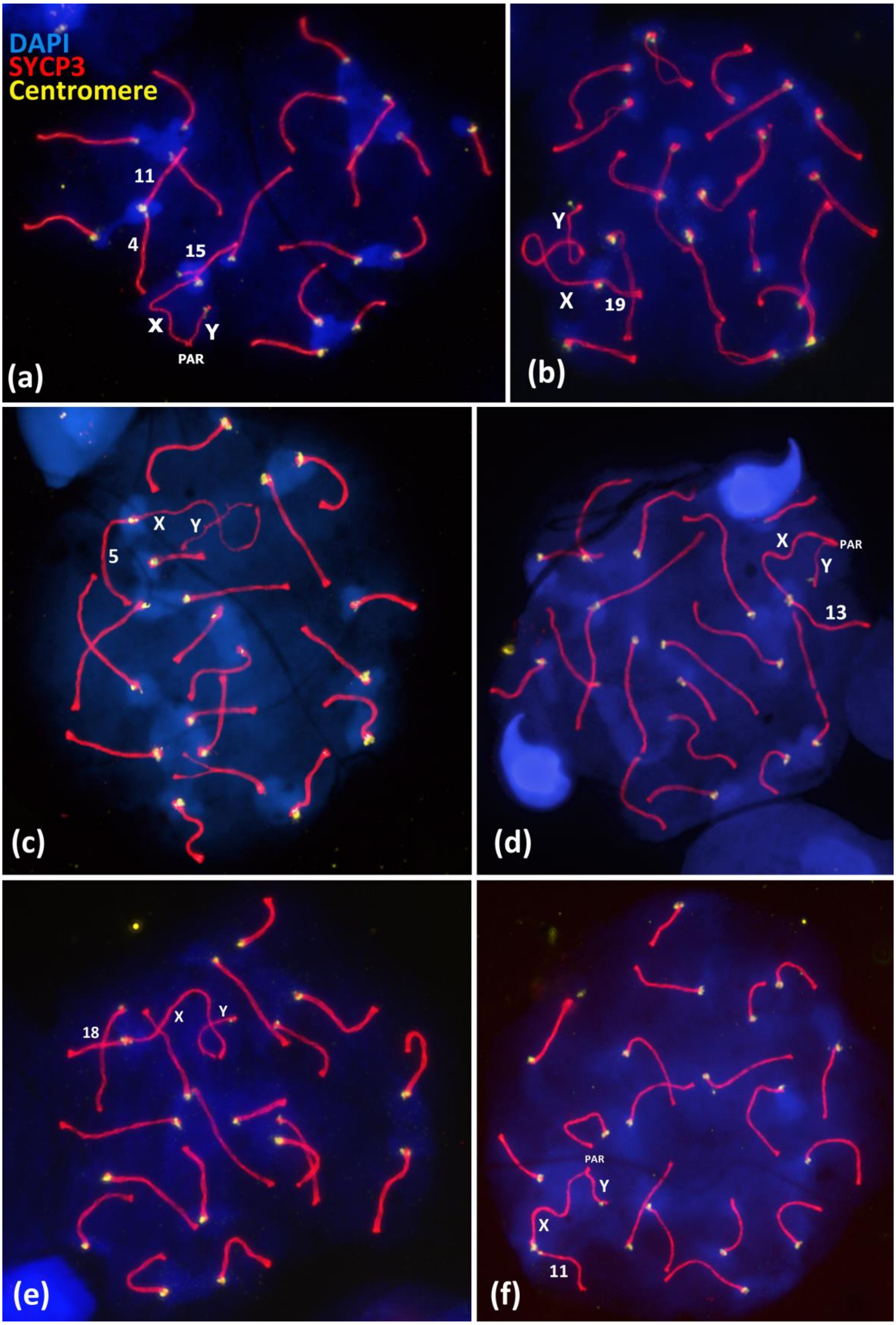

Figure S1. Associations between centromeric regions of autosomal chromosomes and the X chromosome in BALB/c mouse meiotic nuclei **(a-f)**. Chromatin was stained with DAPI (blue), axial elements of meiotic chromosomes were immunostained with the antibodies against the SYCP3 protein (red), centromeres were stained with the ACA antibodies (yellow)

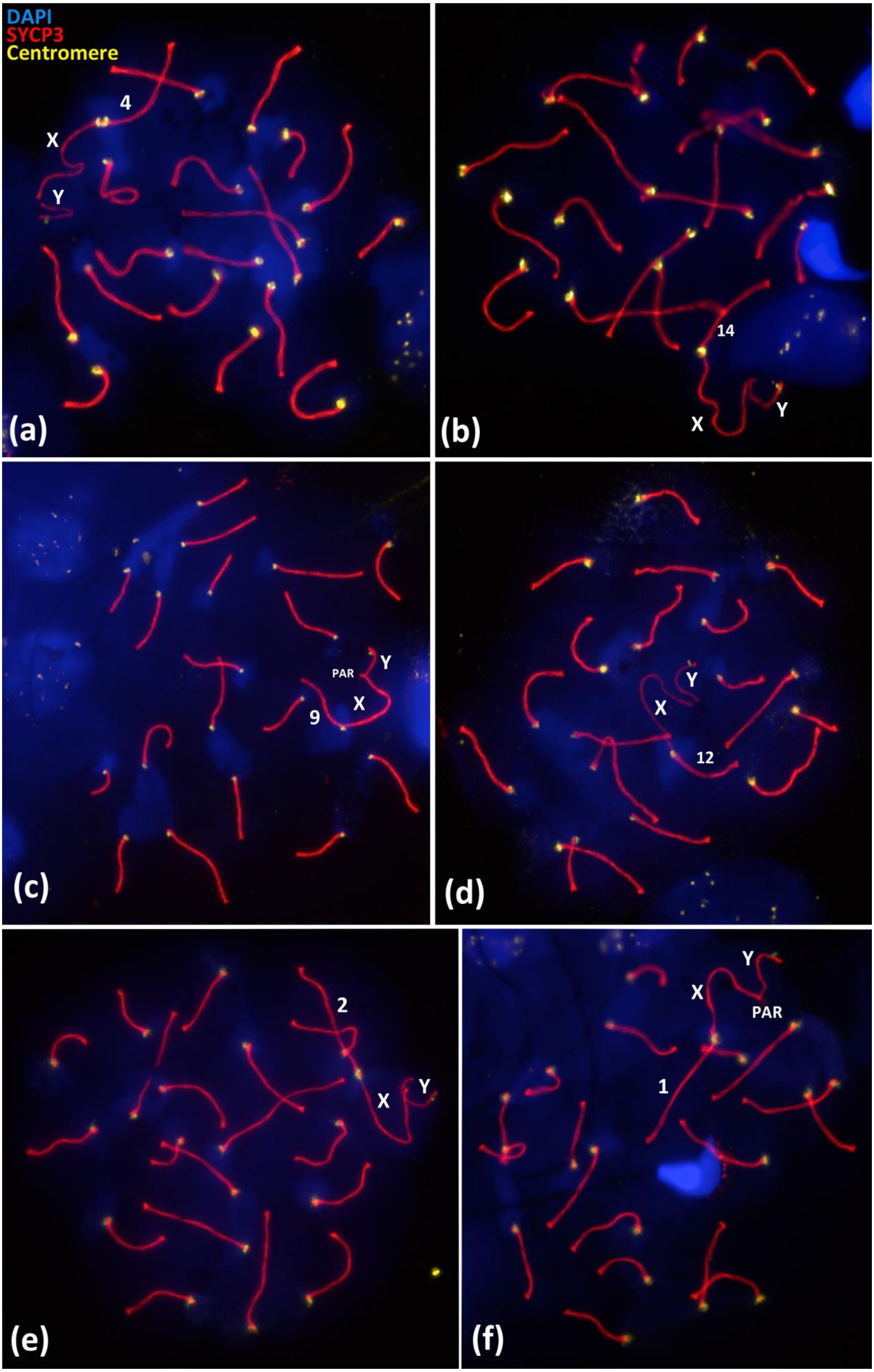

Figure S2. Associations between centromeric regions of autosomal chromosomes and the X chromosome in BALB/c mouse meiotic nuclei **(a-f)**. Chromatin was stained with DAPI (blue), axial elements of meiotic chromosomes were immunostained with the antibodies against the SYCP3 protein (red), centromeres were stained with the ACA antibodies (yellow).

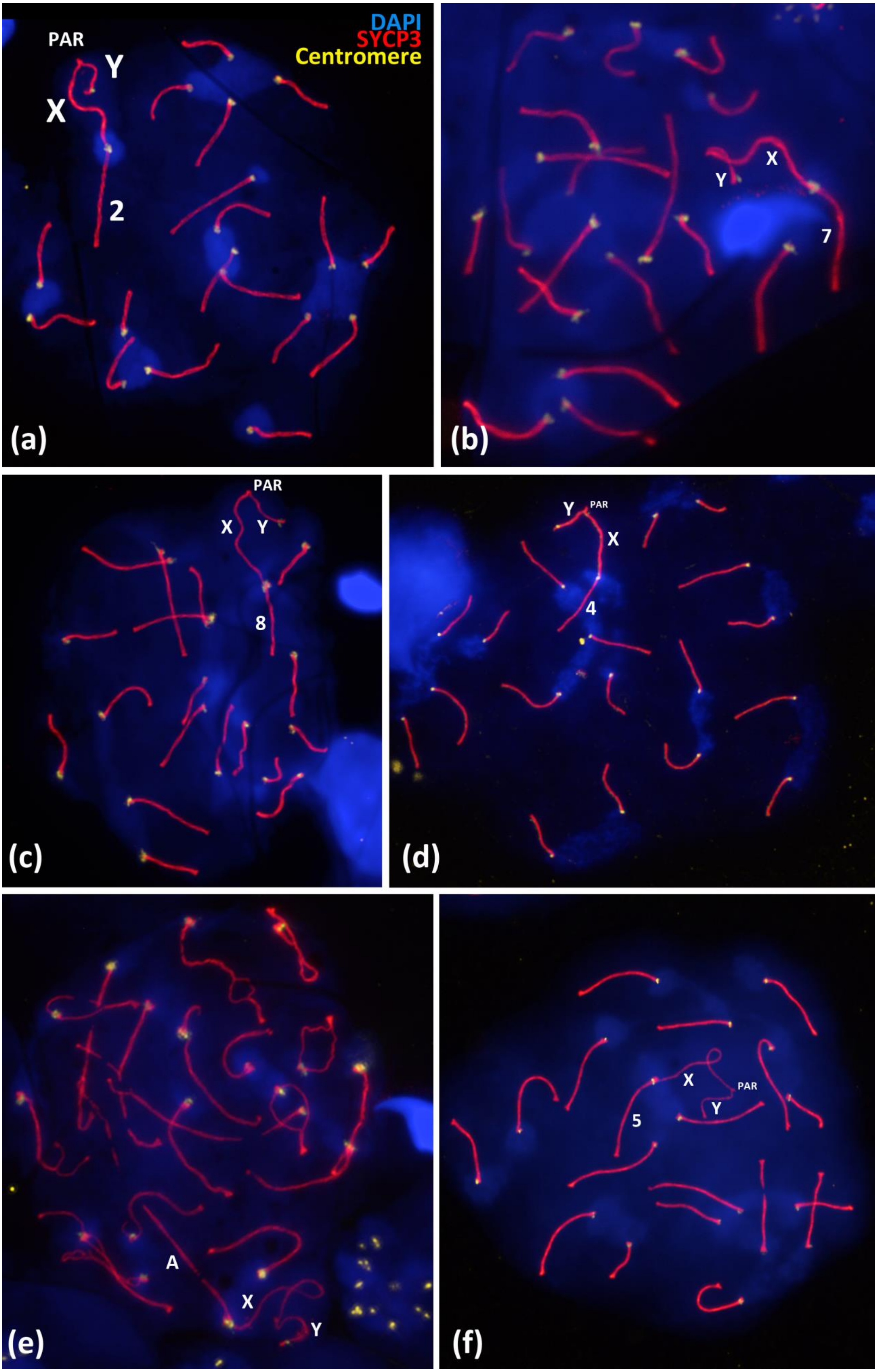

Figure S3. Associations between centromeric regions of autosomal chromosomes and the X chromosome in BALB/c mouse meiotic nuclei **(a-f)**. Chromatin was stained with DAPI (blue), axial elements of meiotic chromosomes were immunostained with the antibodies against the SYCP3 protein (red), centromeres were stained with the ACA antibodies (yellow).

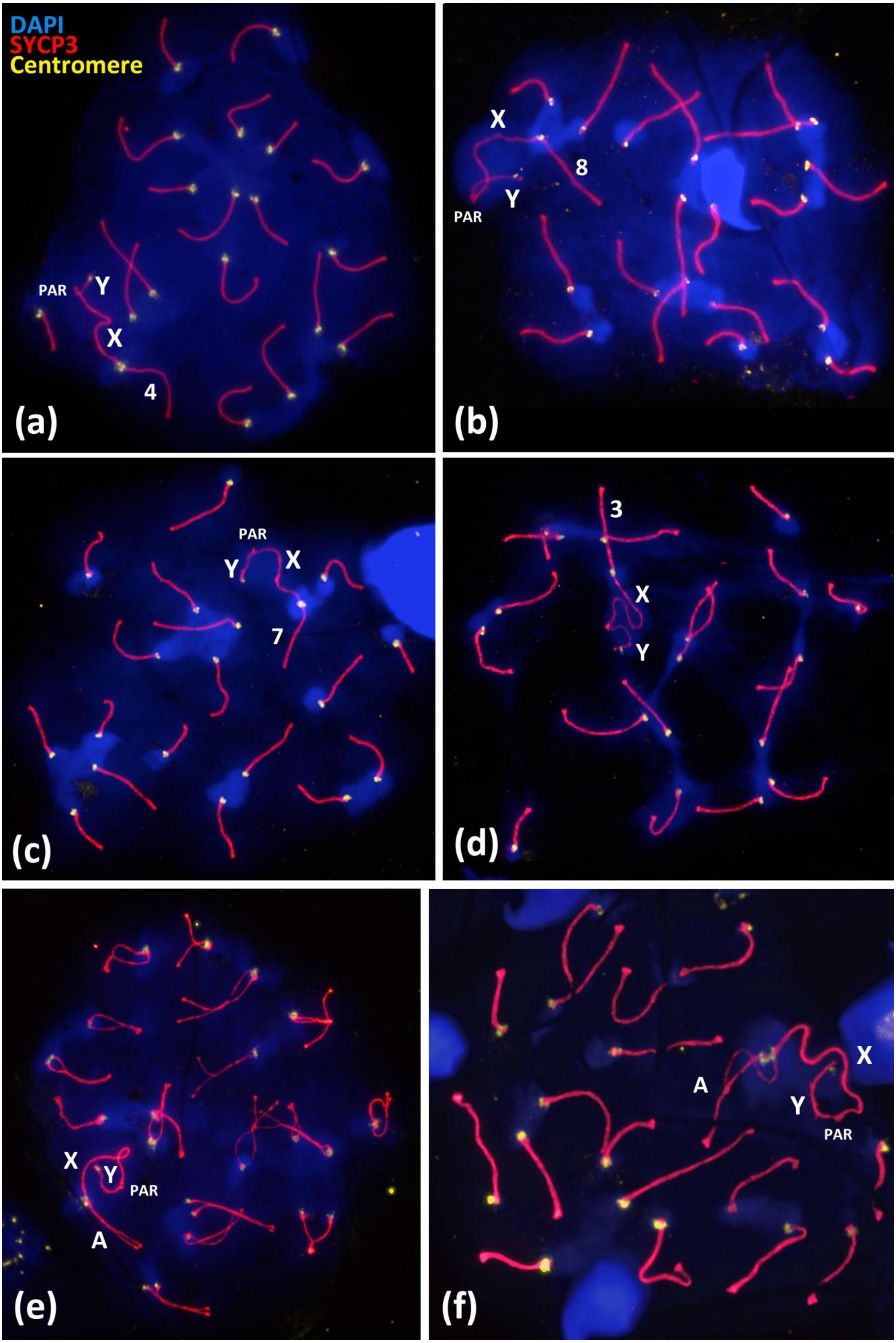

Figure S4. Associations between centromeric regions of autosomal chromosomes and the X chromosome in BALB/c mouse meiotic nuclei **(a-f)**. Chromatin was stained with DAPI (blue), axial elements of meiotic chromosomes were immunostained with the antibodies against the SYCP3 protein (red), centromeres were stained with the ACA antibodies (yellow).

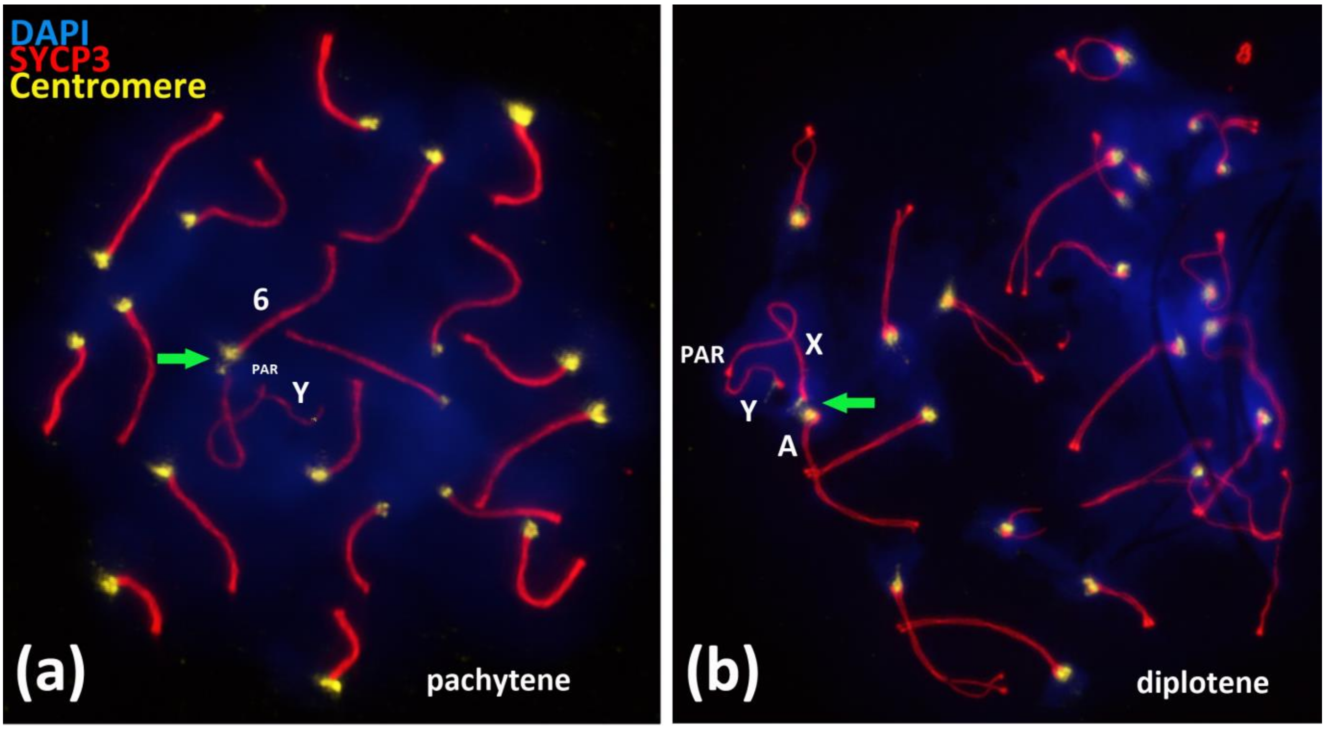

Figure S5. Disrupted associations between centromeric regions of autosomal chromosomes and the X chromosome in BALB/c mouse meiotic nuclei in pachytene **(a)** and diplotene **(b)**. Chromatin was stained with DAPI (blue), axial elements of meiotic chromosomes were immunostained with the antibodies against the SYCP3 protein (red), centromeres were stained with the ACA antibodies (yellow).

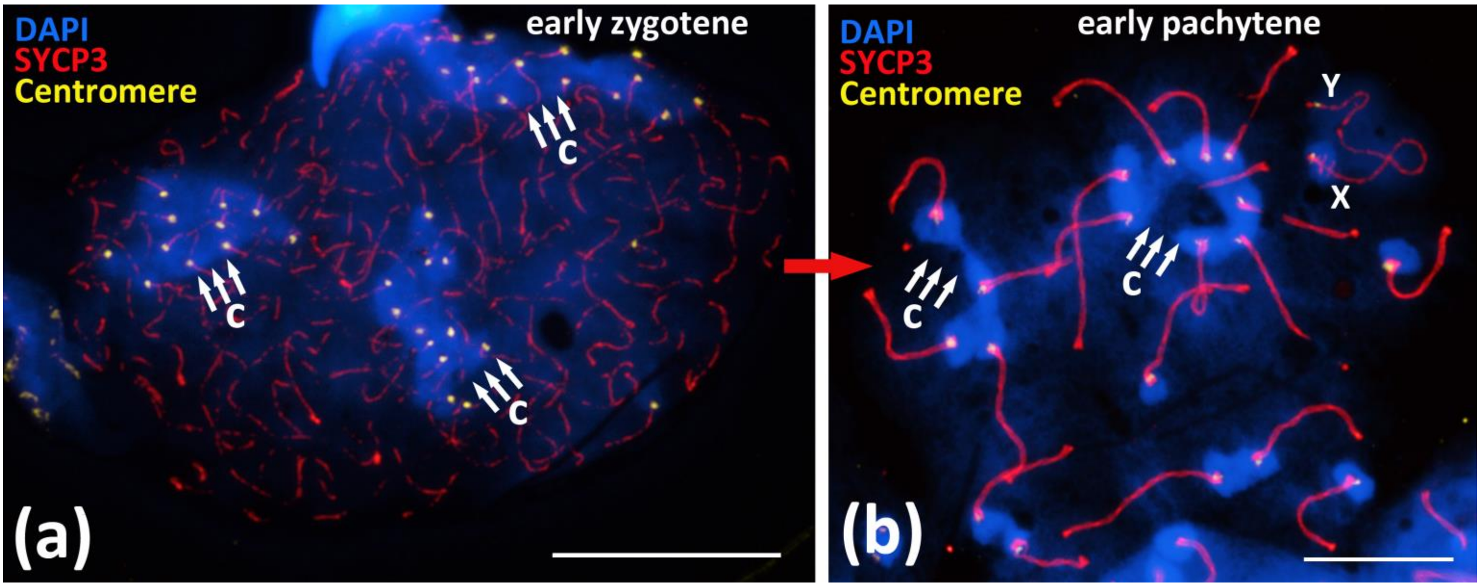

Figure S6. Chromocenters in early zygotene (a) and pachytene (b) in BALB/c mouse. Residual clustering of chromocenters, composed of pericentromeric DAPI-rich regions of bivalents is indicated by triple arrows in the early pachytene nucleus **(b)**. Chromatin was stained with DAPI (blue), axial elements of meiotic chromosomes were immunostained with the antibodies against the SYCP3 protein (red), centromeres were stained with the ACA antibodies (yellow). Scale bar – 10 µm.

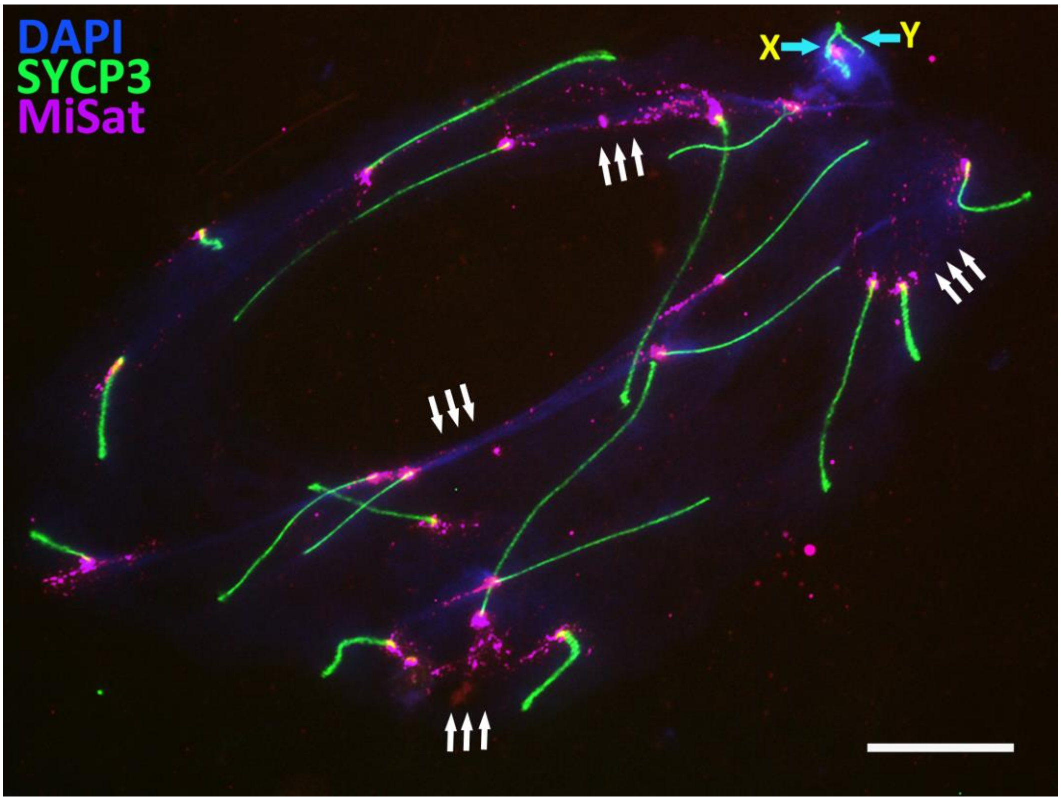

Figure S7. Immuno-FISH with oligo-DNA probes to Minor satellite DNA (MiSat, violet) in the spread preparation of meiotic nucleus BALB/c mouse. X and Y chromosomes are indicated. Chromatin was stained with DAPI (blue), axial elements of meiotic chromosomes were stained with the antibodies against the SYCP3 protein (green). Scale bar – 10 μm.

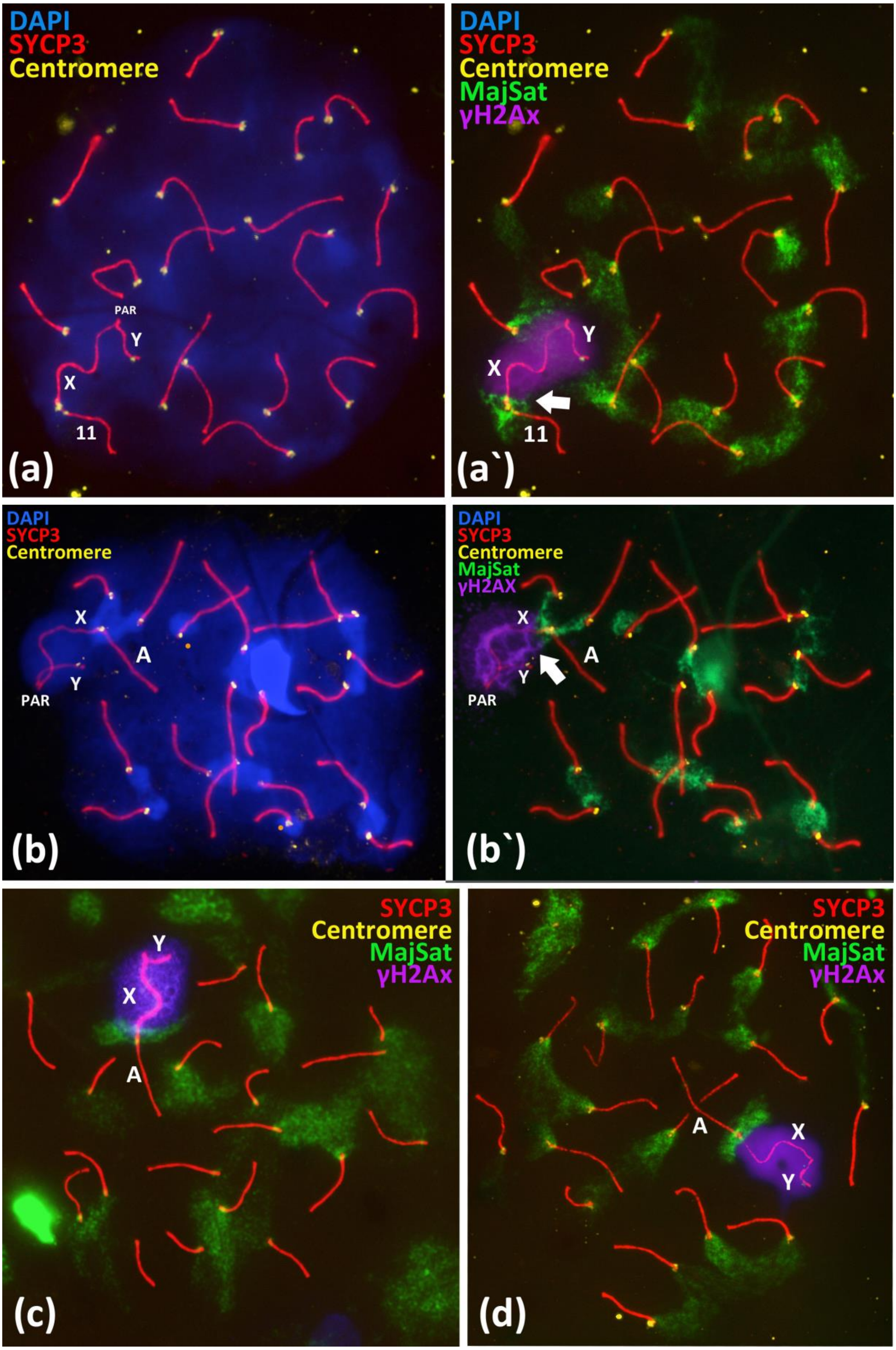

Figure S8. Immuno-FISH identification of MSCI in the associations between Major satellite DNA of the X chromosome and autosomal chromocenters **(a-d)**. Inactivated chromatin of the "sex body" and MajSat DNA are spatially separated **(a',b',c,d)**. Chromatin was stained with DAPI (blue), FISH with oligo-DNA probes to Major Satellite DNA (MajSat, green). Axial elements of meiotic chromosomes were immunostained with the antibodies against the SYCP3 protein (red), centromeres were immunostained with the ACA antibodies (yellow), chromatin of sex bivalent was immunostained with the anti-γH2AX antibodies (violet). Sex chromosomes are indicated "X" and "Y", associated autosomal bivalent – "A".

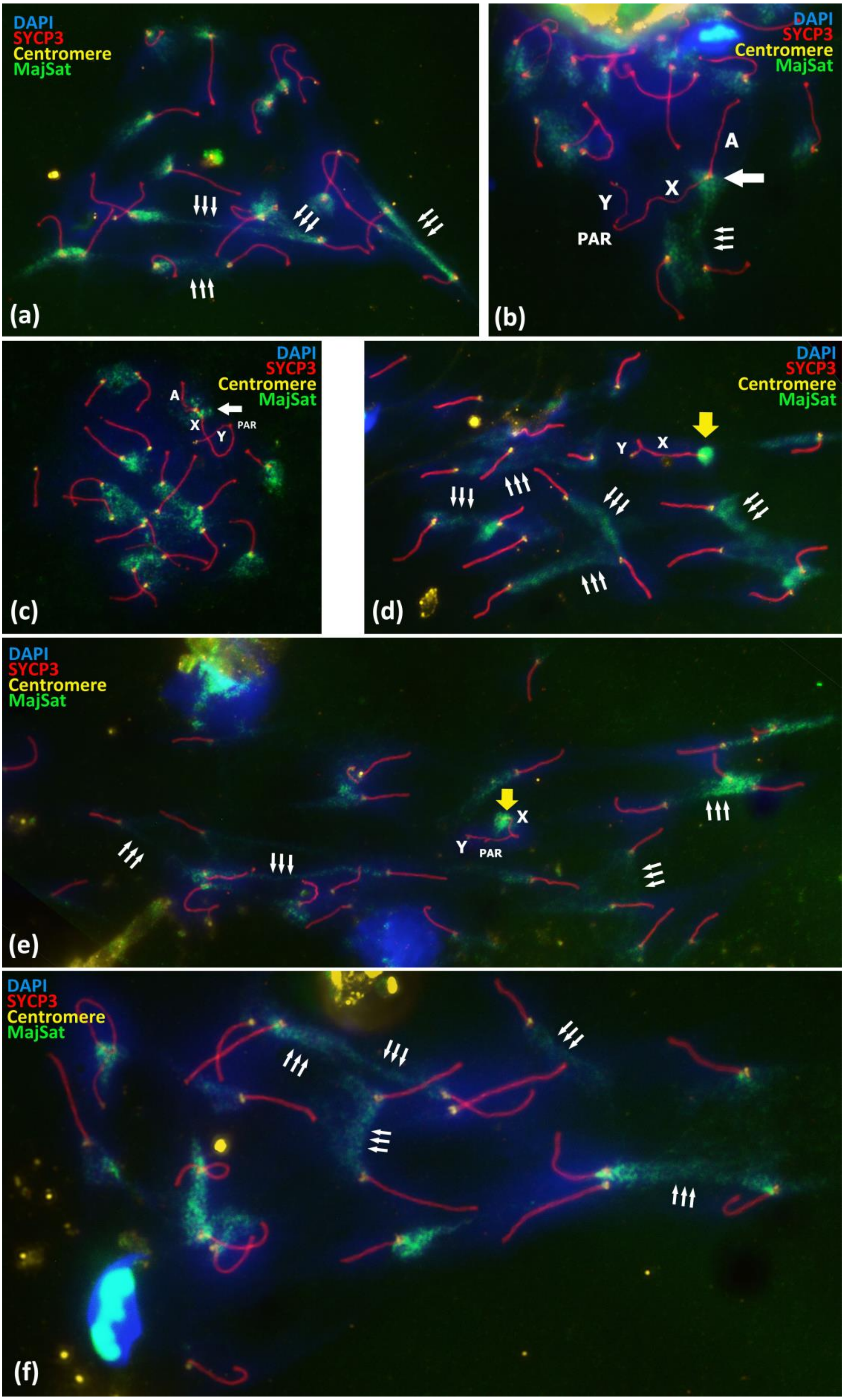

Figure S9. Immuno-FISH study of the Major satellite DNA localization in the pachytene nuclei spread preparations under different spreading conditions, BALB/c mouse. Interbivalent stretched chromatin fibers enriched in MajSat DNA (green) are indicated with triple arrows **(a-f)**. MajSat DNA of X chromosome associated with autosomal chromocenters indicated with single white arrow **(b,c)**. MajSat DNA of X chromosome non-associated with autosomal chromocenters indicated with yellow arrow **(d,e)**.

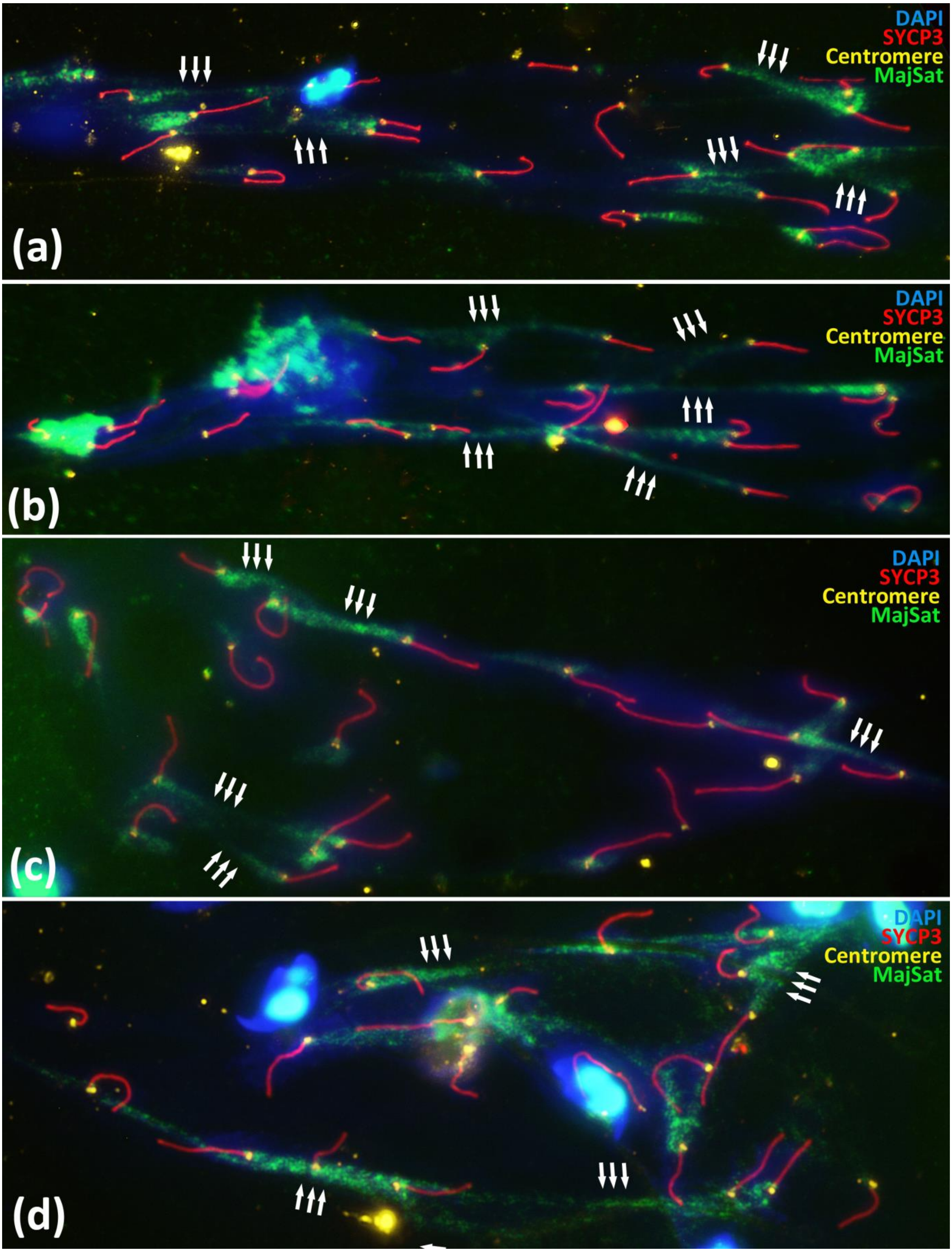

Figure S10. Immuno-FISH study of the Major satellite DNA localization in the pachytene nuclei spread preparations under different spreading conditions, BALB/c mouse. Interbivalental stretched chromatin fibers enriched in MajSat DNA (green) are indicated with triple arrows **(a-d)**.

CBA mouse

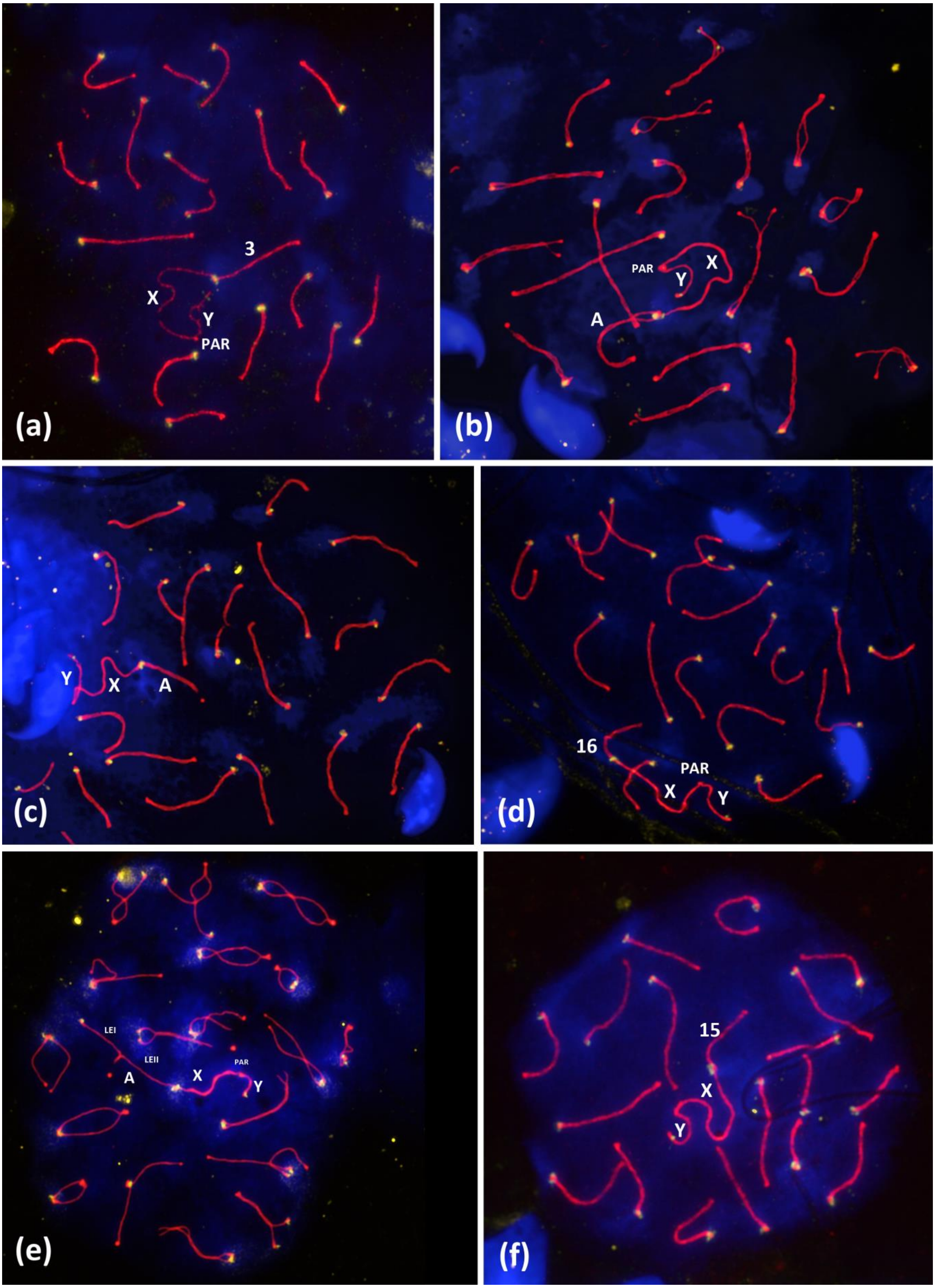

Figure S11. Associations between centromeric regions of autosomal chromosomes and the X chromosome in CBA mouse meiotic nuclei **(a-f)**. Chromatin was stained with DAPI (blue), axial elements of meiotic chromosomes were immunostained with the antibodies against the SYCP3 protein (red), centromeres were stained with the ACA antibodies (yellow).

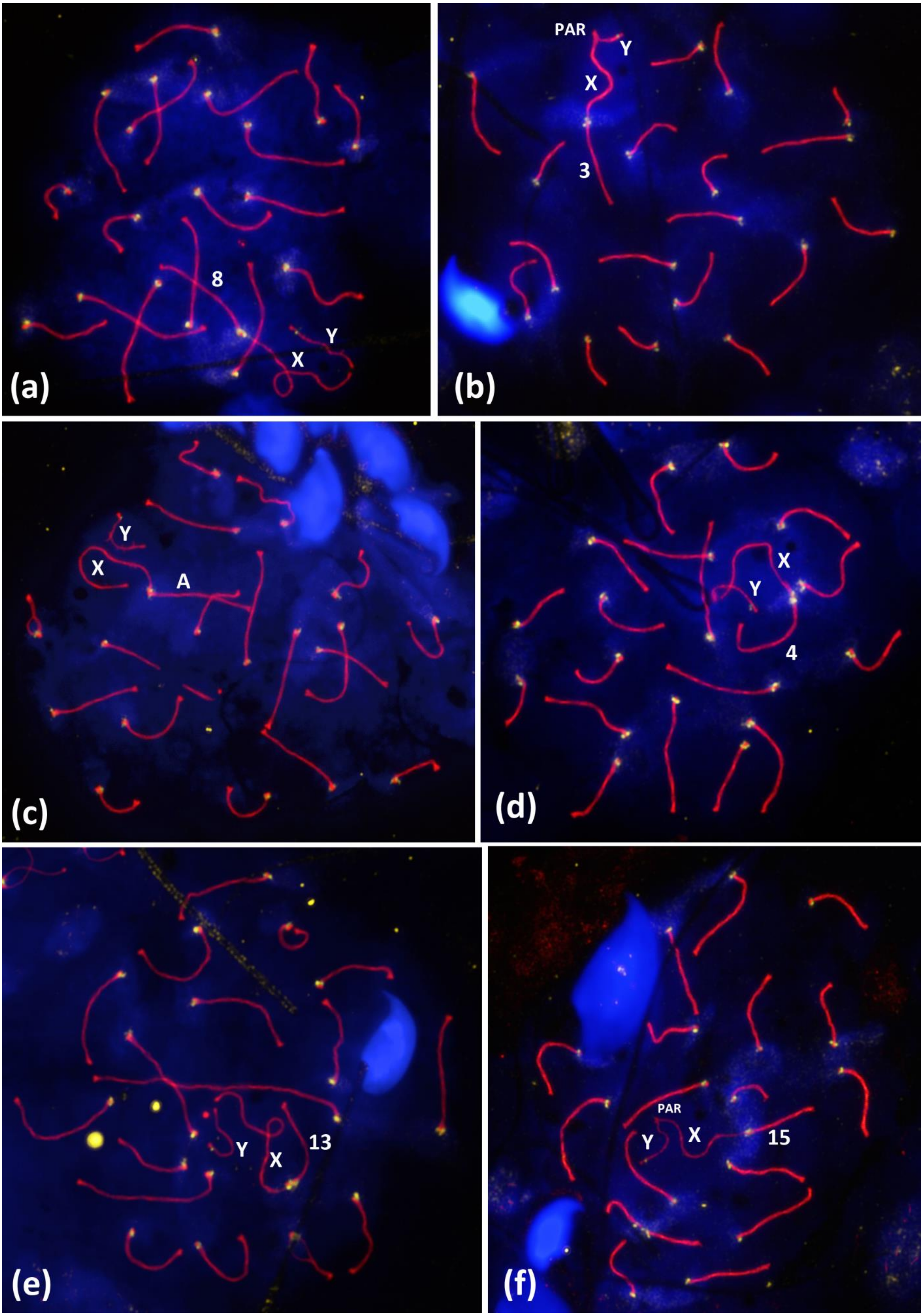

Figure S12. Associations between centromeric regions of autosomal chromosomes and the X chromosome in CBA mouse meiotic nuclei **(a-f)**. Chromatin was stained with DAPI (blue), axial elements of meiotic chromosomes were immunostained with the antibodies against the SYCP3 protein (red), centromeres were stained with the ACA antibodies (yellow).
